# Detection of atrial shunt lesions with a single echocardiographic parameter

**DOI:** 10.1007/s00508-020-01659-0

**Published:** 2020-04-30

**Authors:** Varius Dannenberg, Georg Goliasch, Christian Hengstenberg, Thomas Binder, Harald Gabriel, Matthias Schneider

**Affiliations:** Department of Internal Medicine II, Medical University of Vienna, Waehringer Guertel 18–20, 1090 Vienna, Austria

**Keywords:** RVOT, Right ventricular outflow tract, VTI, Velocity time integral, Atrial septal defect

## Abstract

**Background:**

Unrepaired left to right atrial shunt lesions can cause significant right ventricular (RV) volume overload. The parameter pulmonary to systemic shunt volume ratio (Qp:Qs) has been shown to detect even small differences between left and right ventricular stroke volume; however, four parameters are needed for its calculation. This study was carried out to evaluate the accuracy of the single parameter right ventricular outflow tract (RVOT) velocity time integral (VTI) to identify atrial shunt lesions.

**Methods:**

All patients who underwent transesophageal echocardiography (TEE) examination at this institution between 1 January 2013 and 1 January 2018 were retrospectively analyzed. The RVOT-VTI was measured in the transthoracic echocardiography performed immediately before each TEE. The diagnostic accuracy for detection of atrial shunt lesions was tested.

**Results:**

A total of 2797 patients with a median age of 67 years (interquartile range, IQR 54–77 years) were included in the final analysis. A total of 113 (4%) patients had a relevant atrial shunt lesion. The mean RVOT-VTI of the shunt group was 25 cm (SD ± 8.1 cm) and was significantly higher than that of the non-shunt group with 17 cm (SD ± 4.8 cm) (*p* < 0.001). The area under the curve (AUC) was 0.81. A total of 106 patients (93.8%) of the shunt group had a VTI of ≥16 cm. If the RVOT-VTI was <16 cm, the negative predictive value was 99.3%. If the RVOT-VTI was ≥25 cm, 22% of patients proved to have a significant shunt lesion.

**Conclusion:**

In this large retrospective analysis it could be shown that a low RVOT-VTI predicted the absence of significant atrial shunt lesions, while a high RVOT-VTI predicted the presence. The parameter should be applied in all patients with suspected atrial shunt lesions where calculation of Qp:Qs is impossible.

**Electronic supplementary material:**

The online version of this article (10.1007/s00508-020-01659-0) contains supplementary material, which is available to authorized users.

## Introduction

Unrepaired atrial left to right shunt lesions can lead to significant right heart volume overload. Symptoms such as palpitations, chest pain and shortness of breath are caused by right heart failure and usually occur at a relatively late stage with already dilated right ventricular dimensions and often pulmonary hypertension [1–3]. Therefore, it is crucial to discover early signs of left to right shunts before symptoms develop.

The most common atrial left-to-right shunts include the following defects: ostium secundum atrial septal defect (ASD II), sinus venosus defect (SVD), ostium primum atrial septal defect (ASD I), coronary sinus defect and anomalous pulmonary venous connection [4]. Atrial shunt lesions can occur in combination with a patent foramen ovale (PFO) [5].

Some patients present with electrocardiogram (ECG) changes, such as (in)complete right bundle branch block or they have a fixed split-second heart sound; however, these findings are unspecific [6]. Transthoracic echocardiography (TTE) is the first-line imaging modality applied when there is suspicion of a shunt lesion [7]. Due to low blood flow velocity and the thin interatrial septum, the ASD itself or the shunted blood flow can be missed in TTE [8]. Echocardiographers rely on the presence of secondary signs for right ventricular volume overload, such as right ventricular size and function, presence of tricuspid regurgitation, signs of pulmonary hypertension, and the pulmonary blood flow [7, 9, 10]. For the evaluation of a suspected shunt lesion in TTE, the ratio of the pulmonary to systemic shunt volume (Qp:Qs) is an established method [11, 12]. Due to left-to-right shunting, there is an increased blood flow through the pulmonary circulation in these patients. Kitabatake et al. could show that the Qp:Qs was 0.99 ± 0.05 in healthy subjects [13]. If the ratio is greater than one, a left to right shunt should be considered [14, 15]. The pitfall of this method is the need of four parameters for its calculation: LVOT (left ventricular outflow tract) diameter, RVOT (right ventricular outflow tract) diameter, and the VTI of both outflow tracts (supplementary material 1). Particularly the assessment of the diameters of the outflow tracts can be technically challenging due to image quality [16, 17]. A falsely measured outflow tract diameter leads to wrong assumptions.

We sought to determine if a low VTI can exclude a significant atrial shunt lesion and if a high VTI can be a hint for its presence. RVOT-VTI could be a new diagnostic parameter in TTE for the diagnosis of atrial shunt lesions in those patients where the outflow tract diameters cannot be measured reliably and therefore calculation of Qp:Qs is impossible.

## Methods

Gold standard for the imaging of the interatrial septum and for the diagnosis of atrial shunts is transesophageal echocardiography (TEE) [7]. A retrospective analysis was performed including all patients who received a TEE examination at this institution between 1 January 2013 and 1 January 2018. In addition, a TTE examination (which included a pulsed-wave (PW) Doppler signal in the RVOT) on the same day or in close time interval before the transesophageal echocardiography (TEE) was warranted for inclusion.

The study was conducted in accordance with the amended Declaration of Helsinki. The ethics committee of the Medical University of Vienna approved the conduct of the study (EK# 1427/2018).

The presence of any atrial shunt lesion, the type, and significance were recorded in the defined population. The RVOT VTI of patients without shunt lesions, with non-significant shunt lesions, and with significant shunt lesions were compared. The value of the RVOT VTI for the detection of a significant left to right shunt as a single parameter was evaluated. The analysis was done by postprocessing of ultrasound loops of the cardiology department at the tertiary center.

We excluded all patients with obvious other reasons for high RVOT VTI signals, such as:

- any degree of pulmonary stenosis
- at least moderate pulmonary regurgitation
- more than moderate tricuspid regurgitation

We also excluded those patients with more than mildly reduced right ventricular function (RVF) since there might be false-low RVOT VTI due to reduced cardiac output.

Pulmonary and tricuspid regurgitation were graded by visual quantification of the color Doppler jet. Semiquantitative assessment of RVF was performed by experienced readers using multiple acoustic windows for visual assessment (eyeballing) as well as tissue Doppler imaging of the basal free lateral wall of the RV (S’) and/or tricuspid annular plane systolic excursion (TAPSE), as suggested by the guidelines [18, 19]. The RVF was graded as normal, mildly, moderately, and severely reduced.

### Classification of shunts

A shunt ratio of ≥1.2 for Qp:Qs and/or a shunt diameter of >3 mm were chosen for a shunt to be considered relevant in this study. Few studies have investigated shunt ratios and their impact on the clinical course of patients. From a physiological perspective, a shunt ratio of 1:1 is normal. Based on the data of Engelfriet at al. a ratio of ≥1.5 is considered a relevant shunt which should be considered for surgery/intervention [15, 20]. In this study we did not aim at detecting volume overload in right ventricles but to investigate the feasibility of a new parameter to uncover the presence of a left to right shunt. With 1.2, we therefore chose a ratio below 1.5 and above 1 to define the presence of a shunt lesion.

Statistical methods were independent sample T‑tests and receiver operating characteristics (ROC) curves with area under the curve (AUC). Feasible cut-off values and the Youden index were defined. Sensitivity, specificity as well as positive and negative predictive value were calculated. Regarding optimal VTI cut-off, to find the best diagnostic value for the upper and lower cut-off values for shunt detection and shunt exclusion, we chose the first VTI value with a sensitivity or specificity of >90%. A *p* value ≤0.05 was considered statistically significant. The SPSS Version 24 (SPSS, IBM, Armonk, NY, USA) was used for all analyses.

## Results

**Fig. 1:**
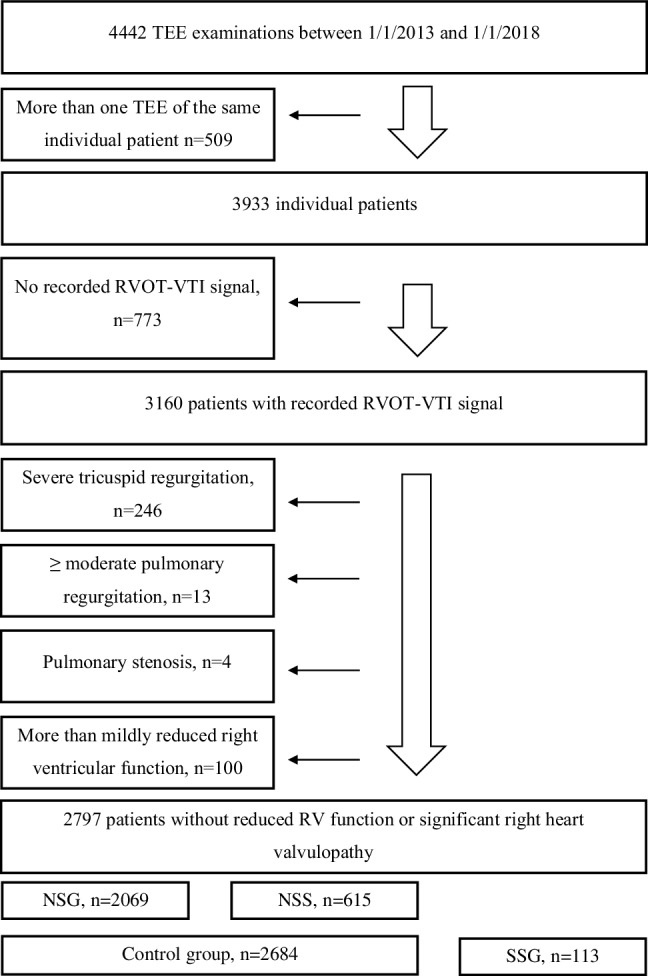
STROBE (strengthening the reporting of observational studies in epidemiology) algorithm showing patient selection. *TEE* transesophageal echocardiography examination, *RVOT* right ventricular outflow tract, *VTI* velocity time integral, *RV* right ventricle, *NSG* no shunt group, *NSS* no significant shunt, *SSG* significant shunt group

Between 1 January 2013 and 1 January 2018 a total of 4442 TEE examinations were performed at this department. If a patient received more than one TEE, we included the first examination and deleted the other(s). All patients with more than mildly reduced right ventricular function as well as those with significant right heart valvulopathy were excluded to allow for unbiased evaluation of RVOT-VTI. A total of 2797 patients (median age 67 years, interquartile range 54–77 years) remained for the final analysis (Fig. 1).

Atrial shunts were found in 728 (26%) patients. This preliminary shunt group was composed of patients with small iatrogenic ASDs after interventions with transseptal puncture (*n* = 27), PFO (*n* = 568), ASD II (*n* = 104), both PFO and ASD II (*n* = 14), ASD I (*n* = 2), and superior SVD (*n* = 13). Inferior sinus venosus ASDs or unroofed coronary sinus defects were not present. In this cohort, there were no patients with ventricular septal defects (VSD) or patent ducts (PDA).

**Fig. 2:**
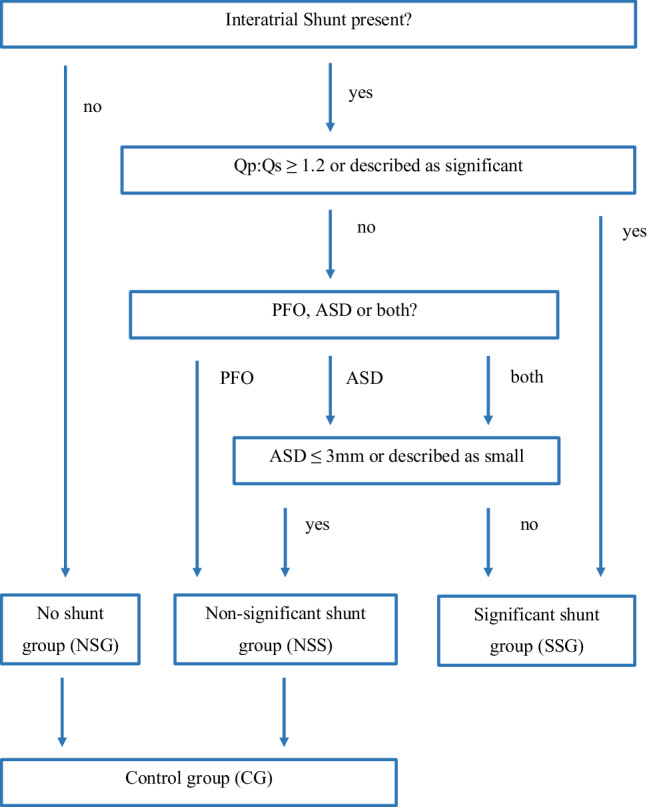
Presence of an interatrial shunt: Qp:Qs indicates the ratio of pulmonary to systemic flow. *Qp:Qs* ratio of pulmonary to systemic flow, *PFO* patent foramen ovale, *ASD* atrial septal defect, *NSG* no shunt group, *NSS* non-significant shunt group; significant shunt group, *CG* control group

Subsequently, all patients without significant shunt lesions were sorted into the non-significant shunt group (NSS). Shunts with Qp:Qs ≥1.2 were directly added to the significant shunt group (SSG). If a Qp:Qs was not reported but the size of the ASD, ASDs ≤3 mm were added to the NSS. A total of 113 patients were assigned to the SSG. Furthermore, 2069 patients without shunts were assigned to the no-shunt group (NSG). The NSG and the NSS together were defined as the control group (CG) (Fig. 2).

Mean RVOT-VTI of the SSG with 25 cm (SD ± 8.1) was significantly higher than that of the CG with 17 cm (SD ± 4.8) (*p* < 0.001) (Table 1).

**Table 1:** Baseline and echo characteristics for significant shunt group and control group

Variable	Significant shunt group	Control group	*p*-value
*Age, years (±SD)*	49.7 (±18.7)	64.7 (±16.0)	**<0.001**
*Female sex, n (%)*	51 (45)	1128 (42)	0.519
*VTI-RVOT, mean (±SD)*	25.04 (±8.1)	17.14 (±4.8)	**<0.001**
*Left ventricular function*			
<moderately reduced LVF, *n* (%)	56 (100)	1427 (94.3)	0.067
≥moderately reduced LVF, *n* (%)	0 (0)	86 (5.7)	0.067
*Right ventricular function*			
Normal RVF, *n* (%)	62 (98.4)	1589 (97.7)	0.718
Mildly reduced RVF, *n* (%)	1 (1.6)	37 (2.3)	0.718
*Right ventricular size*			
Normal sized RV, *n* (%)	26 (40.0)	1685 (97.9)	**<0.001**
Mildly enlarged RV, *n* (%)	18 (27.7)	30 (1.7)	**<0.001**
≥moderately enlarged RV, *n* (%)	21 (32.3)	7 (0.4)	**<0.001**
*Tricuspid valve regurgitation*			
No TR, (%)	0 (0)	21 (1.0)	0.368
Mild TR, *n* (%)	45 (71.4)	1302 (79.0)	0.152
Mild to moderate TR, *n* (%)	12 (19.0)	211 (13.0)	0.148
Moderate TR, *n* (%)	6 (9.5)	115 (7)	0.438

*SD* standard deviation, *VTI* velocity time integral, *RVOT* right ventricular outflow tract, *LVF* left ventricular function, *RVF* right ventricular function, *RV* right ventricle, *TR* tricuspid regurgitation

The ROC curve was calculated for the VTI in all patients, the AUC was 0.819 (95% CI 0.776–0.862). The Youden Index indicated a RVOT-VTI of 21 cm as the value with best sensitivity (72.6%) and specificity (76.8%) for the diagnosis of a significant shunt lesion.

Additional calculations were performed for the extreme cut-offs 16 cm and 25 cm. A total of 106 patients (93.8%) of the SSG had a VTI of ≥16 cm. If VTI was <16 cm, negative predictive value was 99.3%. Only 246 patients (8.8%) had a VTI of ≥25 cm. Of these patients 54 (22%) had a significant shunt lesion (Table 2).

**Table 2:** Diagnostic accuracy of different cut-off values for RVOT-VTI ≥16 cm, ≥21 cm, and ≥25 cm

*VTI ≥16* cm	*SSG*	*CG*	*Total*
Positive	106	1656	1762
Negative	7	1028	1035
Total	113	2684	2797
*VTI ≥21* cm	*SSG*	*CG*	*Total*
Positive	82	623	705
Negative	31	2061	2092
Total	113	2684	2797
*VTI ≥25* cm	*SSG*	*CG*	*Total*
Positive	54	192	246
Negative	59	2492	2551
Total	113	2684	2797

*VTI* velocity time integral, *SSG* significant shunt group, *CG* control group

## Discussion

In this large retrospective study analyzing 2797 patients including 113 with significant atrial shunt lesions, those patients with relevant atrial shunts had a significantly higher RVOT-VTI than those without a shunt lesion. Therefore, RVOT-VTI should be applied as a single parameter in those patients, where Qp:Qs calculation is impossible due to image quality.

Any unexplained dilatation of the right heart chambers must raise suspicion for the presence of significant atrial shunt lesions. Careful TTE imaging of the interatrial septum via 2D echocardiography from atypical views, the application of color Doppler imaging as well as intravenous contrast agents help with the diagnosis. In cases with limited image quality, the morphology of the interatrial septum can remain inconclusive in TTE. Due to large shunt areas and therefore slow blood flow through the lesion, color Doppler imaging can be inconclusive as well. In these patients, significant blood flow from left to right can inhibit right heart contrast agent from crossing to the left heart. The typical wash-out phenomenon can be very clear via TEE but can be difficult to detect in TTE. This may leave echocardiographers with the isolated finding of a dilated right heart and no further information on the possible presence of a shunt lesion. With Qp:Qs, a reliable echocardiographic hemodynamic parameter is available for further evaluation and for the decision making if the particular patient is referred to TEE, where a definitive diagnosis can be made. Unfortunately, especially the correct measurement of the diameter of the RVOT is impossible in numerous patients due to image quality. At the same time, RVOT-VTI can be measured in almost all patients in the parasternal long or short axis, in the apical inflow-outflow view, or from subcostal angulation without the need for good image quality. In this analysis, we sought to evaluate if the single parameter RVOT-VTI can distinguish between patients with and without atrial shunt lesions.

With a mean of 25 cm and 17 cm respectively, RVOT-VTI was significantly (*p* < 0.001) higher in patients with relevant atrial shunt lesions than in those without. In ROC analysis, AUC to differentiate between the two groups was 0.819. The Youden index determined a VTI of 21 cm as the best value regarding specificity and sensitivity; however, neither specificity (76.8%) nor sensitivity (72.6%) are good enough to use RVOT-VTI of 21 cm as diagnostic guidance in clinical practice. We therefore analyzed the extreme cut-offs at the lower and the upper end of the VTI spectrum.

Considering a VTI of 16 cm, only 7 of the 113 significant shunt lesions fell below this cut-off, negative predictive value was 99.3%. It can be concluded that a significant shunt lesion is unlikely if VTI is <16 cm.

The RVOT-VTI values of patients with an ASD I or a superior SVD lesion were high with 31.5 cm and 31.8 cm, respectively. In those patients with a VTI of 25 cm and more, 22% of patients proved to have a significant shunt lesion. In the light of these data, a high RVOT-VTI signal in combination with a dilated right heart should prompt the suspicion for the presence of a significant atrial shunt lesion, if other reasons for high RVOT-VTI signals have been previously ruled out. A TEE should be carried out as a further diagnostic test in these patients.

Apart from the validation of RVOT-VTI as a diagnostic parameter for the detection of shunt lesions, this work also allows a closer look at the prevalence of shunt lesions at this tertiary center in Austria. The analysis includes all TEE examinations between 1 January 2013 and 1 January 2018. In total, 113 significant shunt lesions were found. In this adult cohort with a median age of 67 years, ASD II was the most frequent shunt lesion with 79% of cases, followed by superior SVD in 11.5% of cases.

Finally, the age of the patients was investigated and the mean age in the significant shunt group was 49 years. This shows that atrial shunt lesions are firstly diagnosed even in older patients and can exist over a long time without symptoms. Thus, significant atrial shunt lesions are an important differential diagnosis not only in young patients. Every effort must be made by the echocardiographer to find the underlying disease if an enlarged right heart is detected.

For future studies, corrections for clinical states with high cardiac output would be of interest. This could be done by measuring the VTI of the LVOT and comparing it with the VTI of the RVOT. This parameter would be one step closer to Qp:Qs, but without the error prone measurements of the outflow tracts.

### Limitations

The retrospective design of this study has several limitations. The exact position of the PW-Doppler sample volume could not be evaluated retrospectively. A different position of the sample volume in the pulmonary artery or in the distal RVOT can change the value. Clinical situations with high cardiac output such as pregnancy, fever, hyperthyroidism, anemia, and dialysis shunts are expected to present with high VTIs without presence of an atrial shunt lesion. Due to lack of clinical data and the retrospective design of the study, these patients could not be excluded from the analysis.

The bias of these limitations was reduced by choosing a longer period of time and thus a larger number of patients.

## Conclusion

In this large retrospective analysis, we could show that a low RVOT-VTI predicted the absence, while a high RVOT-VTI predicted the presence of significant atrial shunt lesions. The parameter should be applied in all patients with suspicion for atrial shunt lesions where calculation of Qp:Qs is impossible.

## Caption Electronic Supplementary Material

This supplementary depicts the necessary measurements for Qp:Qs calculation. Panel A (RVOT) and Panel C (LVOT) show the outflow tract measurements. In Panel B (RVOT) and Panel D (LVOT) VTI measurements are shown. Qp:Qs indicates the ratio of pulmonary to systemic flow. If both ventricles have the same stroke volume, the ratio is 1.0. *RVOT* right ventricular outflow tract; *VTI* velocity time integral; *LVOT* left ventricular outflow tract
