# Supplementary material for: Detection of atrial shunt lesions with a single echocardiographic parameter
Source: Wien Klin Wochenschr. 2020 Apr 30;132(11):295–300. doi: 10.1007/s00508-020-01659-0 (PMC7297847; doi:10.1007/s00508-020-01659-0)

Supplementary material 1. *Qp:Qs measurement. Qp:Qs indicates the ratio of pulmonary to systemic flow. If both ventricles have the same stroke volume, the ratio is 1.0.*

RVOT, right ventricular outflow tract; VTI, velocity time integral; LVOT, left ventricular outflow tract.

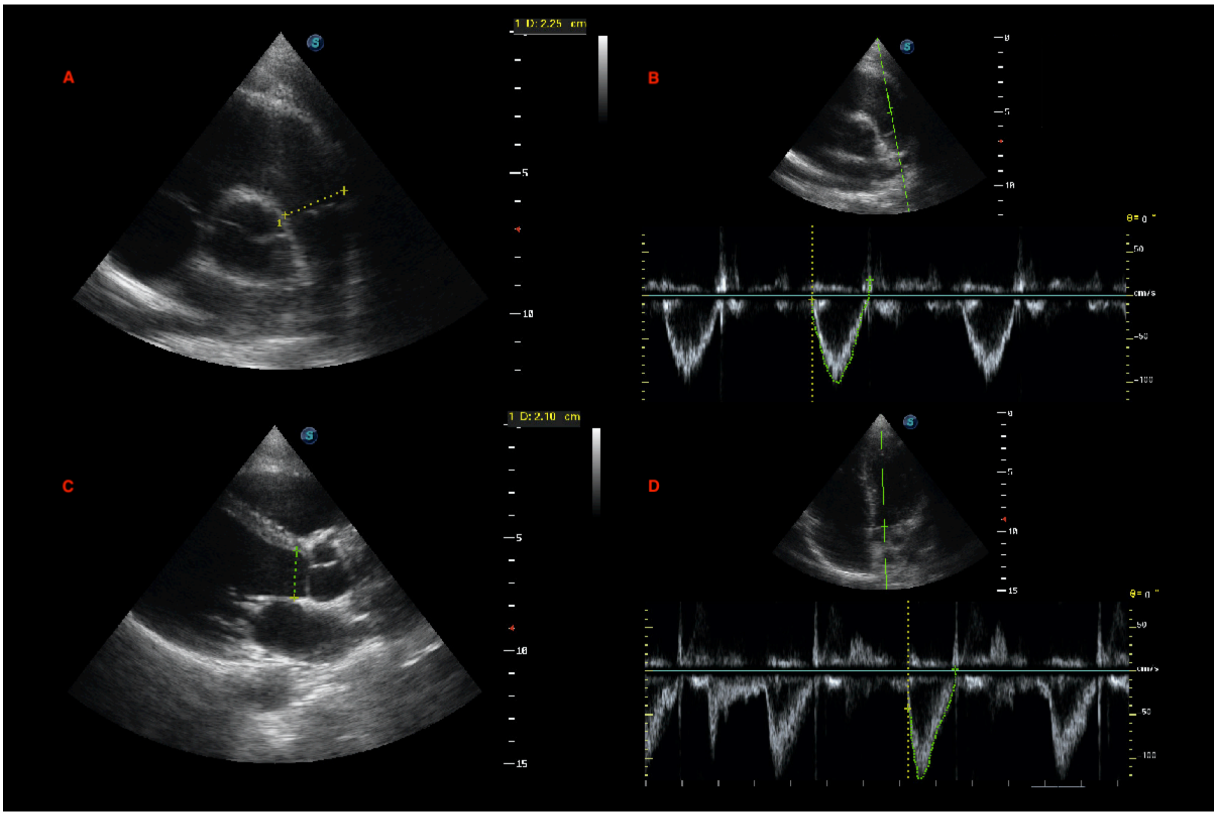

Supplement: Supplementary file 1 — This supplementary depicts the necessary measurements for Qp:Qs calculation. Panel A (RVOT) and Panel C (LVOT) show the outflow tract measurements. In Panel B (RVOT) and Panel D (LVOT) VTI measurements are shown. Qp:Qs indicates the ratio of pulmonary to systemic flow. If both ventricles have the same stroke volume, the ratio is 1.0. RVOT right ventricular outflow tract; VTI velocity time integral; LVOT left ventricular outflow tract [file 508_2020_1659_MOESM1_ESM.pdf]
